# The distribution of porphyrins with different tumour localising ability among human plasma proteins.

**DOI:** 10.1038/bjc.1989.38

**Published:** 1989-02

**Authors:** M. Kongshaug, J. Moan, S. B. Brown

**Affiliations:** Institute for Cancer Research, Norwegian Radium Hospital, Oslo.

## Abstract

The distribution among the main fractions of human plasma lipoproteins of a number of porphyrins with different tumour localising ability has been determined by means of ultracentrifugation. A main trend is that the fraction of the dyes that are bound to low density lipoprotein (LDL) increases, and the fraction bound to HSA decreases with decreasing polarity of the dyes. An asymmetric charge distribution, such as in TPPS2a, favours LDL-binding more than expected on the basis of lipophilicity. No correlation between the known tumour localising ability of the drugs tested in the present work and their relative affinity for LDL was found. One of the best tumour localisers reported in the literature, TPPS4, hardly binds to LDL, while Hp and Pp, which are commonly considered inefficient tumour localisers, do have a significant affinity for LDL. On the other hand, the LDL binding capacity for a drug is suggested to be a good index for cellular uptake. Such an index does not necessarily imply that the actual uptake occurs by the LDL pathway.


Br. J. Cancer (1989), 59, 184–188 © The Macmillan Press Ltd., 1989

### The distribution of porphyrins with different tumour localising ability among human plasma proteins

M. Kongshaug[1], J. Moan[1] & S.B. Brown[2]

[1]*Institute for Cancer Research, The Norwegian Radium Hospital, Montebello, Oslo 3, Norway; and* [2]*University of Leeds, UK.*

**Summary** The distribution among the main fractions of human plasma lipoproteins of a number of porphyrins with different tumour localising ability has been determined by means of ultracentrifugation. A main trend is that the fraction of the dyes that are bound to low density lipoprotein (LDL) increases, and the fraction bound to HSA decreases with decreasing polarity of the dyes. An asymmetric charge distribution, such as in $TPPS_{2a}$, favours LDL-binding more than expected on the basis of lipophilicity. No correlation between the known tumour localising ability of the drugs tested in the present work and their relative affinity for LDL was found. One of the best tumour localisers reported in the literature, $TPPS_4$, hardly binds to LDL, while Hp and Pp, which are commonly considered inefficient tumour localisers, do have a significant affinity for LDL. On the other hand, the LDL binding capacity for a drug is suggested to be a good index for cellular uptake. Such an index does not necessarily imply that the actual uptake occurs by the LDL pathway.

Subsequent to studies describing porphyrin binding to plasma lipoproteins (Jori *et al.*, 1984; Reyftman *et al.*, 1984; Moan *et al.*, 1985), including the observation that lipoproteins play an important role in the transport of haematoporphyrin (Hp) in the bloodstream (Jori *et al.*, 1984), it has been suggested that the tumour localising property of the porphyrin photosensitisers used in photodynamic therapy (PDT) is related to such binding, particularly to low density lipoprotein (LDL) (Reyftman *et al.*, 1984; Barel *et al.*, 1986; Kessel, 1986; Kessel *et al.*, 1987). The possibility of delivering various kinds of cytotoxic, antitumoral and/or photosensitising drugs via the receptor-mediated pathway of LDL has received much attention because various types of cancer cells have considerably higher LDL receptor activity than the corresponding normal cells (e.g. Norata *et al.*, 1984; Vitols *et al.*, 1985; Gal *et al.*, 1981; Iwanik *et al.*, 1984). Such enhanced LDL activity is supposedly due to the high requirement for cholesterol in rapidly proliferating cells, LDL being the major extracellular vehicle for the transport of cholesterol to extrahepatic animal and human cells (Brown *et al.*, 1981).

Following administration of haematoporphyrin derivatives (HpD) to patients (D.I. Vernon, unpublished) or to experimental animals, the major tumour localising fraction preferentially binds to LDL and HDL (Kessel, 1986). This fraction of HpD was not detected at all in high performance liquid chromatography (HPLC) analysis of the albumin fraction of the mouse plasma, neither 0.5 h nor 48 h after the administration of HpD. The binding of Hp and HpD to lipoproteins may influence their distribution in different tissues. Thus, administration to tumour-bearing mice of *in vitro* prepared complexes of Hp and LDL appears to deliver substantially more Hp to the mouse tumour (MS-2 fibrosarcoma) than does administration of Hp-complexes of very low density lipoproteins (VLDL), high density lipoproteins (HDL) or free Hp (Barel *et al.*, 1986).

In the present work we have studied the pattern of distribution among human plasma proteins of a range of porphyrin photosensitisers relevant to PDT. These compounds vary significantly, both with respect to lipophilicity and, in certain cases, their known uptake and tumour selectivity. (The latter two terms are here defined as by Peng *et al.* (1987).) Our objectives were to find out if there is any correlation between the lipophilicity (determined by HPLC-retention times) and the LDL-binding capacity for these drugs and to discuss whether such capacity may be a main determinant for tumour localisation.

Correspondence: J. Moan.
Received 24 June 1988, and in revised form, 26 September 1988.

It has been reported that some PDT sensitisers are cleared more rapidly from LDL than from HDL in mice, implying that there is no equilibrium redistribution (Barel *et al.*, 1986; Kessel, 1986). We have checked if this can be explained by a slow redistribution of drugs among the different plasma proteins.

### Materials and methods

### *Materials*

Hp, protoporphyrin (Pp) and photoprotoporphyrin (Ppp) were bought from Porphyrin Products (Logan, UT, USA). PhotofrinII (PII) ($2.5\,mg\,ml^{-1}$) was obtained from Photomedica (Raritan, NJ, USA) and stored frozen in small vials. The tetraphenyl porphine sulphonates ($TPPS_{1-4}$) were kind gifts from Dr Bruce Burnham at Porphyrin Products. These sulphonated porphyrins belong to the same batch as that used by Kessel *et al.* (1987), and were tested chromatographically by that group. Their chromatographic analysis agreed with our own (Figure 4). Stock solutions of Hp, PII, $TPPS_3$ and $TPPS_4$ were made up in 0.03 M NaOH + 0.15 M NaCl, whereas dimethyl sulphoxid (DMSO) was stock solvent for Pp, Ppp, $TPPS_{2o}$ and $TPPS_{2a}$.

All other chemicals used were of the highest purity commercially available.

Fresh human plasma sampled in EDTA tubes was used in all experiments and was obtained from the same healthy person who had fasted overnight. All solutions for ultracentrifugation were made up with 0.1 M Tris-HCl buffer, pH 7.35, containing 0.4 mg EDTA (potassium salt) per ml.

### *Ultracentrifugation*

A Beckman L8-70M ultracentrifuge with a fixed angle 70.1 Ti rotor was used. It was found that a 15 h run at 70,000 r.p.m. was suitable for a good separation of the different protein fractions. A 74 h run at 32,000 r.p.m., using a centrifuge with an SW-40 swinging bucket rotor, gave similar results, the only significant difference being a slightly better separation at the top of the gradient (i.e. in the VLDL/LDL region). The high-speed/short time method was used in this study, to reduce ultracentrifuge time and to reduce possible ageing effects on the samples. Our gradient, as well as those used by others (Chapman *et al.*, 1981; Kelly & Krusky, 1986), is slightly modified from that used by Redgrave *et al.* (1975). The top fraction of the gradient was made up using 0.15 M NaCl, but at higher densities we employed CsCl instead of NaCl/KBr, as CsCl gives a lower molarity of salt at a given density. This fact may be important for the separation since it is well known that an increase in the salt concentration results in a decrease in porphyrin solubility. Furthermore, for the same reason we chose to apply the drug-containing plasma at the top of the gradient instead of at $d = 1.15$–$1.312\,g\,ml^{-1}$ as employed by other groups (Chapman *et al.*, 1981; Nilsson *et al.*, 1981; Kelly & Krusky, 1986; Redgrave *et al.*, 1975). Pure plasma gives a similar protein distribution pattern whether it was applied at the top or at the bottom of the gradient. Finally, we chose to use a density of $1.35\,g\,ml^{-1}$ at the bottom of the tube (Figure 1), instead of 1.21, to prevent the heaviest proteins and/or protein–porphyrin complexes from precipitating at the bottom. Several gradients known from the literature (Chapman *et al.*, 1981; Kelly & Krusky, 1986; Nilsson *et al.*, 1981) were tested in a series of experiments using the SW-40 rotor and found to give similar relative separations as the gradient used by us. A separate paper, giving the explicit experimental data on which our choice of method is based, will be published later.

*Concentration*

The porphyrin binding patterns obtained here refer to $7\,\mu g\,ml^{-1}$ plasma in the case of PII and $14\,\mu g\,ml^{-1}$ plasma in the case of all other porphyrins. Reducing the plasma concentration by a factor of 5 while keeping the total porphyrin concentration constant (i.e. 35–70 $\mu g$ porphyrin per ml plasma equivalent to 2.5–5 mg per kg bodyweight) gave similar results as for 7–14 $\mu g\,ml^{-1}$ for all substances tested in this respect, i.e. Hp, PII, Pp and $TPPS_4$.

*Analysis*

Gradients are often analysed from the bottom up. It was found that this method resulted in a contamination of the HDL fraction by other heavy proteins and an apparatus was therefore constructed which analysed and fractionated the gradient from the top downwards. By means of a peristaltic pump and an LKB 2098 Uvicord III monitor with appropriate optical filters the absorbences (1 cm) at 276 nm (proteins) and at 405 nm (porphyrins) were continuously monitored. Fractions were also collected and measured spectrophotometrically (Cary 118) since the tetraphenyl porphine sulphonates have their absorption maximum at 417 nm in our solvent. From one sample to another the position of the protein maxima in the gradient might change slightly (i.e. by $\pm$ one fraction). However, with three exceptions (see **Results**) the maxima of the porphyrin distribution coincided exactly with the maxima of the protein distribution in the same run.

*HPLC*

High performance liquid chromatography was carried out with an RP18 column and a methanol/water gradient buffered at pH 7.4 as described previously (Sommer *et al.*, 1984).

### Results

The initial gradient and that obtained after centrifugation are shown in Figure 1. The protein distribution ($A_{280nm}$), is shown in the upper parts of Figures 2 and 3. LDL, lipoprotein(a) (Lp(a)), HDL and heavy proteins (mostly human serum albumin (HSA)) are well separated. The separation is similar to the separations obtained by others by the use of lower speeds and longer times of centrifugation, except that in our high r.p.m. experiments VLDL is not resolved from the LDL peak. Using the present gradient, but lower centrifugation rates (SW-40 rotor) and longer times, the VLDL was separated fropm the LDL peak (data not shown). From such data it may be inferred that 5–15% of the LDL-area in Figures 2 and 3 is due to VLDL. The reproducibility from one run to another and between blood

**Figure 1** Outline of the gradient before (left) and after centrifugation (15 h, 70,000 r.p.m.). The classification of the lipoprotein is according to Patch & Patch (1986).

**Figure 2** (a) Protein distribution in the gradient. Mean value and standard errors from six individual samples. (b) Distribution in the gradient of the following porphyrins: $TPPS_1$ (○), $TPPS_{2a}$ (△), $TPPS_{2o}$ (▽), $TPPS_3$ (□) and $TPPS_4$ (×). Reproducibility between separate runs better than 10% of the values shown (in the LDL region, the errors are larger (20%) in the case of low LDL binding).

**Figure 3** (a) Protein distribution in the gradient. (b) Distribution in the gradient of the following porphyrins: Pp (△), Hp (○) and Ppp (□). (c) Distribution of PII in the gradient. Centrifugation: 15 h, 70,000 r.p.m.

**Figure 4** (a) HPLC of a mixture of $TPPS_4$, $TPPS_3$, $TPPS_{2o}$, $TPPS_{2a}$ and $TPPS_1$ on a RP 18 column eluted with a water/methanol gradient. The porphyrins were also run separately for identification of the peaks. (b) Distribution of the same porphyrins between LDL, HDL and heavy proteins (HSA on the figure). The percentage bound to each fraction was determined by weighing of distributions such as those shown in Figure 2. LDL, fractions 1–11; HDL, fractions 11–23; HSA, fractions 23–32.

**Table I** Distribution of porphyrins among human plasma proteins (centrifugation 15 h, 15°C, 70,000 r.p.m.)

| *Porphyrin* | *HPLC ret. time (min) on RP18* | *Distribution (%)* LDL | HDL | Heavy proteins |
|---|---|---|---|---|
| Ppp | 9.5 | 9 | 57 | 34 |
| Hp | 3.6, 4.0 | 10 (6.5) | 55 (38.4) | 35 (55.1) |
| PII | 3.6–20 | 16 | 70 | 14 |
| Pp | 18 | 22 (20) | 41 (38) | 37 (42) |
| $TPPS_4$ | 0.05 | 1–2 (3) | 18 (14) | 80–81 (81) |
| $TPPS_3$ | 0.35 | 6 (6) | 68 (18) | 26 (73) |
| $TPPS_{2o}$ | 3.95 | 7 (17) | 74 (43) | 19 (38) |
| $TPPS_{2a}$ | 10.1 | 36 (38) | 55 (53) | 9 (5) |
| $TPPS_1$ | 20.0 | 30 (23) | 60 (80) | 10 (<2) |

Data from Reyftman *et al.* (1984) for Hp and Pp and Kessel *et al.* (1987) for the TPPS-series are included in parentheses for comparison.

Reproducibility of percentages bound to the different proteins better than 15% of the values shown, cf. legend of Figure 2.

samples taken on different days was good, consistent with the s.e. bars shown on Figure 2.The maxima of the LDL, Lp(a), HDL and HSA peaks were found at fractions 4–5, 11–13, 17–19 and 28–29, respectively (Figures 2 and 3). In the cases of Hp, Pp, Ppp, PII, TPPS, $TPPS_1$ and $TPPS_{2a}$ (Figures 1 and 2), the maxima of the dye distribution always coincide with the maxima of the protein peaks. However, in the case of $TPPS_3$ the 417 nm peak in the HDL-region was shifted by one fraction to the right compared to the 280 nm peak and in the case of $TPPS_{2o}$ the 417 nm peak in the HDL-region was shifted by three fractions to the right compared to the 280 nm peak. The latter shift is seen in the lower part of Figure 2. Furthermore, the major peak of the $TPPS_4$-adduct(s) is slightly shifted to the denser region of the heavy proteins by about one fraction, as seen in Figure 2.

Integration over distributions, as shown in Figures 2 and 3 by weighing the areas under the curves between given fractions (see legend to Figure 3), resulted in the data shown in Table I. Thus, in the present work we have not attempted to measure thermodynamic affinities but only the relative distributions of the drugs between the plasma proteins. We emphasise that the concentration of each plasma protein in the samples has remained constant to within 5% throughout the work. There may be a slightly different distribution of aggregated and monomeric species among the different serum proteins. However, absorbance measurements as performed in the present work appear to give correct distributions since fluorescence measurement was found to give similar distributions (data not shown). (The fluorescence quantum yields of porphyrins are very sensitive to the state of aggregation.) The porphyrin amount bound to VLDL was small compared to that bound to LDL (less than 15%) and therefore these two fractions are taken together. Only Pp and $TPPS_{2a}$ showed a tendency to bind to Lp(a). Even in these two cases, the fraction of the porphyrins bound to Lp(a) is small compared to that bound to HDL (< 5%) and these two fractions are taken together in Table I.

When PII, Hp or Pp were incubated with plasma for different times (0, 4 and 16 h) at 37°C their distributions were almost identical to those shown in Figure 3.

### Discussion

The relative binding of the porphyrins to LDL increases generally, as expected, with increasing lipophilicity (Table I, Figure 3). Thus, Pp is more extensively bound to LDL than the structurally similar, but more polar Hp. Correspondingly, the relative binding of the porphyrins to the heavy proteins (mainly HSA) generally decreases with increasing lipophilicity, as best illustrated by the TPPS-series (Table I, Figure 3). However, there are important exceptions to this general trend: Ppp and Hp bind similarly in the heavy protein region in spite of the fact that the latter porphyrin is significantly more polar than the former one. Similarly $TPPS_{2a}$ binds more extensively to LDL than does $TPPS_1$, which is significantly less polar. This may be related to the asymmetric charge distribution on $TPPS_{2a}$, which may cause a high affinity for a lipid/water interface. The asymmetry of $TPPS_{2a}$ has been previously invoked by Kessel *et al.* (1987) as an explanation for their observation that $TPPS_{2a}$ has a higher uptake in cells than $TPPS_1$. In summary, the relative binding capacity LDL has for a drug is mainly related to the lipophilicity of the drug, although other factors, such as properties of different sidegroups and the asymmetry/symmetry of the charge distribution play important roles for this affinity.

For most of the drugs we have tested, where these can be compared with results in the literature, there is generally broad agreement (Table I). However, in the cases of Hp and $TPPS_2$ we find relatively less in the heavy fraction and in the LDL fraction and more in the HLD fraction than did Reyftman *et al.* (1984) and Kessel *et al.* (1987). The discrepancy is even greater in the case of $TPPS_3$, for which we find much more in the HDL fraction and much less in the heavy protein fraction than did Kessel *et al.* (1987). The porphyrins which we find mainly in the heavier part of HDL, i.e. in $HDL_3$ (see Figure 1), seem in Kessel's experiments to have been less well resolved from the albumin peak than in our case. This may be related to an effect of salt on the relative binding of $TPPS_3$ as in his experiments the porphyrin-labelled plasma was applied at a high KBr concentration. Indeed, we have shown (unpublished results) that, in the case of Hp, high salt causes more Hp to bind in the heavy pattern region. The fact that $TPPS_3$ and $TPPS_{2o}$ seem to be bound more substantially to the heavier subfraction of HDL than the other porphyrins tested should be noted. These compounds are more polar than $TPPS_{2a}$ and $TPPS_1$, just as the heavier subfraction of HDL is more polar than its lighter fraction. It is also noteworthy that the $TPPS_4$-peak at high densities seems to occur at a somewhat higher density than the protein peak in that region, implying that $TPPS_4$ appears to be bound not only to albumin but also to one or more of the other heavy proteins.

HpD injected in mice is reported to be lost much faster from the LDL fraction than from the HDL fraction (Kessel, 1986), as is Hp injected in mice and rabbits (Barel *et al.*, 1986). Consequently, these observations would seem to indicate that the binding and release of these drugs to the lipoproteins is slow and that LDL is degraded faster than HDL. The same group showed for systemic administration of Hp to cancer patients that, although Hp is lost with similar rates from LDL and HDL, it is lost at lower rates from VLDL (Jori *et al.*, 1984). However, from the present results the distributions of PII as well as those of Hp and Pp between the plasma proteins are similar for different incubation times ranging from 0 to 4 h (PII) and 0 to 16 h (Hp and Pp) at 37°C (data not shown). Thus equilibrium seems to be rapidly reached. Similarly, recent results obtained by ultracentrifugation of sera from patients injected with PII indicate that there is no change in the distribution of the drug with time between injection and sampling (Brown *et al.*, work in progress), which is in agreement with our present results of rapid establishment of equilibrium. The possibility that there is a species difference in the relative stability of circulating porphyrin–lipoprotein complexes should be noted (but see below).

The assumption to be found in the literature that the tumour localising ability of porphyrins used in PDT may be related to their relative binding to LDL (Jori *et al.*, 1984; Reyftman *et al.*, 1984; Barel *et al.*, 1986; Kessel, 1986) is not fully supported by the present work. Hp has a higher relative affinity for LDL than $TPPS_4$ and Pp has an even higher affinity (Table I), but Hp and Pp are generally considered inefficient tumour localisers. PII has a relative affinity for LDL lying between that of Hp and that of Pp but is a good tumour localiser as reported by a number of authors (see the review by Moan, 1986). Moreover, $TPPS_4$ has a very low affinity for LDL (see Table I) and a relatively high affinity for heavy proteins, but is still one of the best tumour localisers studied so far, with respect both to absolute tumour uptake and to selectivity (Winkleman, 1985; Evensen, 1985; Peng *et al.*, 1987). According to Kessel *et al.* (1987), albumin-bound drugs accumulate preferentially in stromal elements in tumour tissue while lipoprotein-bound drugs mediate intracellular localisation. As noted above, $TPPS_4$ appears to be bound not only to albumin but also to other heavy proteins which may contribute to its distribution *in vivo*.

The tumour selectivity of a drug may be related as much to its retention in tumour tissue as to its initial deposition. For this initial deposition, LDL transport may play an important role. Thus, it has been shown that a lipophilic fluorophore carried by LDL is selectively deposited in endothelial cells in the vasculature (Netland *et al.*, 1985). Furthermore, strong PDT-effects on endothelial cells have been reported (Chaudhuri *et al.*, 1987). The difference in retention of different porphyrins in cells is demonstrated by our HPLC experiments, showing that washing of HpD-loaded cells with a medium containing serum results in a selective removal of the monomeric components Hp, Pp and hydroxyethylvinyl deuteroporpohyrin (Moan & Sommer, 1983).

Quite apart from the actual mechanisms of the drug uptake, there exists the possibility that the binding capacity for a drug (rather than its lipophilicity) is a good index for cellular uptake, as suggested by previous results for cellular uptake of TPPS analogues (Kessel *et al.*, 1987).

We are forced to conclude that at present the determinants of tumour localisation of different drugs are poorly understood. LDL-binding may play one role, heavy proteins another. Other lipoproteins, such as VLDL, HDL ($HDL_2$ and/or $HDL_3$) and perhaps VHDL (very high density lipoproteins) as well as other factors such as aggregation properties, change in polarity with pH (Moan *et al.*, 1980) chemical nature of side groups or the presence of different metal ligands (Hambright *et al.*, 1975; Winkelman, 1967) may also be important.

We thank the Norwegian Cancer Society and the Yorkshire Cancer Research Campaign for financial support.

### References

BAREL, A., JORI, G., PERIN, P., PAGNAN, A. & BIFFANTI, S. (1986). Role of high-, low- and very low-density lipoproteins in the transport and tumor-delivery of hematoporphyrin *in vivo*. *Cancer Lett.*, **32,** 145.

BROWN, S.M., KOVANEN, P.T. & GOLDSTEIN, J.L. (1981). Regulation of plasma cholesterol by lipoprotein receptors. *Science*, **212,** 628.

CHAPMAN, M.J., GOLDSTEIN, S., LAGRANGE, D. & LAPLAUD, P.M. (1981). A density gradient ultracentrifugal procedure for the isolation of the major lipoprotein classes from human serum. *J. Lipid Res.*, **22,** 339.

CHAUDHURI, K., KECK, R.W. & SELMAN, S.H. (1987). Morphological changes of tumor microvasculature following hematoporphyrin derivative sensitized photodynamic therapy. *Photochem. Photobiol.*, **46,** 823.

EVENSEN, J.F. (1985). Distribution of tetraphenyl sulphonate in mice bearing Lewis lung carcinoma. In *Photodynamic Therapy of Tumors and Other Diseases*, Jori, G. & Perria, C. (eds) p. 215. Libreria Progetto: Padova.

GAL, D., McDONALD, P.C., PORTER, J.C. & SIMPSON, E.R. (1981). Cholesterol metabolism in cancer cells in monolayer culture: III. Low density lipoprotein metabolism. *Int. J. Cancer*, **29,** 315.

HAMBRIGHT. P., FAWWAZ, R., VALK, P., McRAE, J. & BEARDEN, A.J. (1975). The distribution of various watersoluble radioactive metalloporphyrins in tumorbearing mice. *Bioinorganic Chem.*, **5**, 87.

IWANIK, M.J., SHAW, K.V., LEDWITH, B.J., KANOVICH. S. & SHAW, J.M. (1984). Preparation and interaction of a low-density lipoprotein: Daunomycin complex with P388 leukemic cells. *Cancer Res.*, **44**, 1206.

JORI, G., BELTRAMINI, M., REDDI, E. & 4 others (1984). Evidence for a major role of plasma lipoproteins as hematoporphyrin carries *in vivo*. *Cancer Lett.*, **24**, 291.

KELLY, J.L. & KRUSKI, A.W. (1986). Density gradient ultracentrifugation of serum lipoproteins in a swinging bucket rotor. In: *Methods in Enzymology, Vol. 128*, Segrest, J.P. & Albers, J.J. (eds) p. 170. Academic Press: New York.

KESSEL, D. (1986). Porphyrin-lipoprotein association as a factor in porphyrin localization. *Cancer Lett.*, **33**, 183.

KESSEL, D., THOMPSON, P., SAATIO, K. & NANTWI, K.D. (1987). Tumor localization and photosensitization by sulfonated derivatives of tetraphenylporphine. *Photochem. Photobiol.*, **45**, 787.

MOAN, J. (1986). Porphyrin photosensitization and phototherapy. *Photochem. Photobiol.*, **43**, 681.

MOAN, J., RIMINGTON, C., EVENSEN, J. & WESTERN, A. (1985). Binding of porphyrins to serum proteins. In *Methods in Porphyrin Photosensitization*, Kessel, D. (ed) p. 193. Plenum Press: New York.

MOAN, J., SMEDSHAMMER, L. & CHRISTENSEN, T. (1980). Photodynamic effects on human cells exposed to light in the presence of hematoporphyrin. pH effects. *Cancer Lett.*, **9**, 327.

MOAN, J. & SOMMER, S. (1983). Uptake of the components of hematoporphyrin derivative by cells and tumors. *Cancer Lett.*, **21**, 167.

NETLAND, P.A., BRUCE, R.Z., VIA, D.P. & VOYTA, J.C. (1985). *In situ* labelling of vascular endothelium with fluorescent acetylated low density lipoproteins. *Histochem. J.*, **17**, 1309.

NILSSON, J., MANNIKAROTTEN, V., EDELSTEIN, C. & SCANU, A.M. (1981). An improved detection system applied to the study of lipoproteins after singlestep density gradient ultracentrifugation. *Anal. Biochem.*, **110**, 342.

NORATA, G., CAUTI, G., RICCI, L., NICOLIN, A., TREZZI, E. & CATOPANO, A.L. (1984). *In vitro* assimilation of low density lipoproteins by a fibrosarcoma tumor line in mice. *Cancer Lett.*, **25**, 203.

PATCH, J.R. & PATCH, W. (1986). Zonal ultracentrifugation. In *Methods in Enzymology, vol. 129*, Albers, J.J. & Segrest, J.P. (eds). Academic Press: New York.

PENG, Q. EVENSEN, J.F., RIMINGTON, C. & MOAN, J. (1987). A comparison of different photosensitizing dyes with respect to uptake in C3H-tumors and tissues of mice. *Cancer Lett.*, **36**, 1.

REDGRAVE, T.G., ROBERTS, C.K. & WEST, C.E. (1975). Separation of plasma lipoproteins by density gradient ultracentrifugation. *Anal. Biochem.*, **65**, 42.

REYFTMAN, J.P., MORLIERE, P., GOLDSTEIN, S., SANTUS, R., DUBERTRET, L. & LAGRANGE, D. (1984). Interaction of human serum low density lipoproteins with porphyrins: a spectroscopic and photochemical study. *Photochem. Photobiol.*, **40**, 721.

RUDLING, M.J., COLLINS, V.P. & PETERSON, C.O. (1983). Delivery of oclacinomycin A to human glioma cells *in vitro* by the low-density lipoprotein pathway. *Cancer Res.*, **43**, 4600.

SOMMER, S., MOAN, J., CHRISTENSEN, T. & EVENSEN, J. (1984). A chromatographic study of hematoporphyrin derivatives. In *Porphyrins in Tumor Phototherapy*, Andreoni, A. & Cubeddu, R. (eds) p. 81. Plenum Press: New York.

VITOLS, S., GATRON, G. & PETERSON, C. (1984). Significance of the low-density lipoprotein (LDL) receptor pathway for the *in vitro* accumulation of AD-32 incorporated into LDL in normal and leukemic white blood cells. *Cancer Treat. Rep.*, **68**, 515.

VITOLS, S.G., MASQUELIER, M. & PETERSON, C.O. (1985). Selective uptake of a toxic lipophilic anthrocycline derivative by the low density lipoprotein receptor in cultured fibroblasts. *J. Med. Chem.*, **2**, 451.

WINKELMAN, J. (1967). Metabolic studies of the accumulation of tetraphenylporphine sulfonate in tumors. *Experientia*, **23**, 949.

WINKELMAN, J.W. (1985). Quantitative studies of tetraphenylporphine sulfonate and hematoporphyrin derivative distributions in animal tumor systems. In *Methods in Porphyrin Photosensitization*, Kessel, D. (ed) p. 91. Plenum Press: New York.


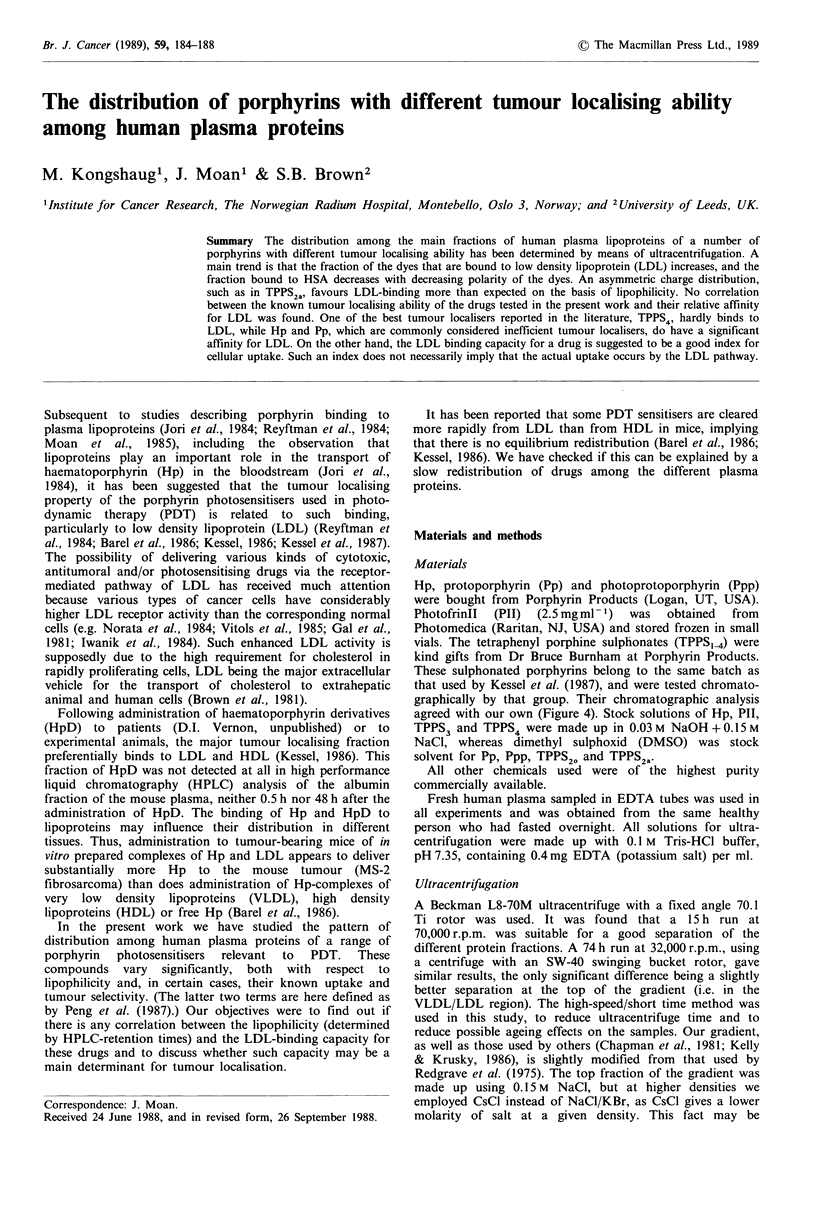

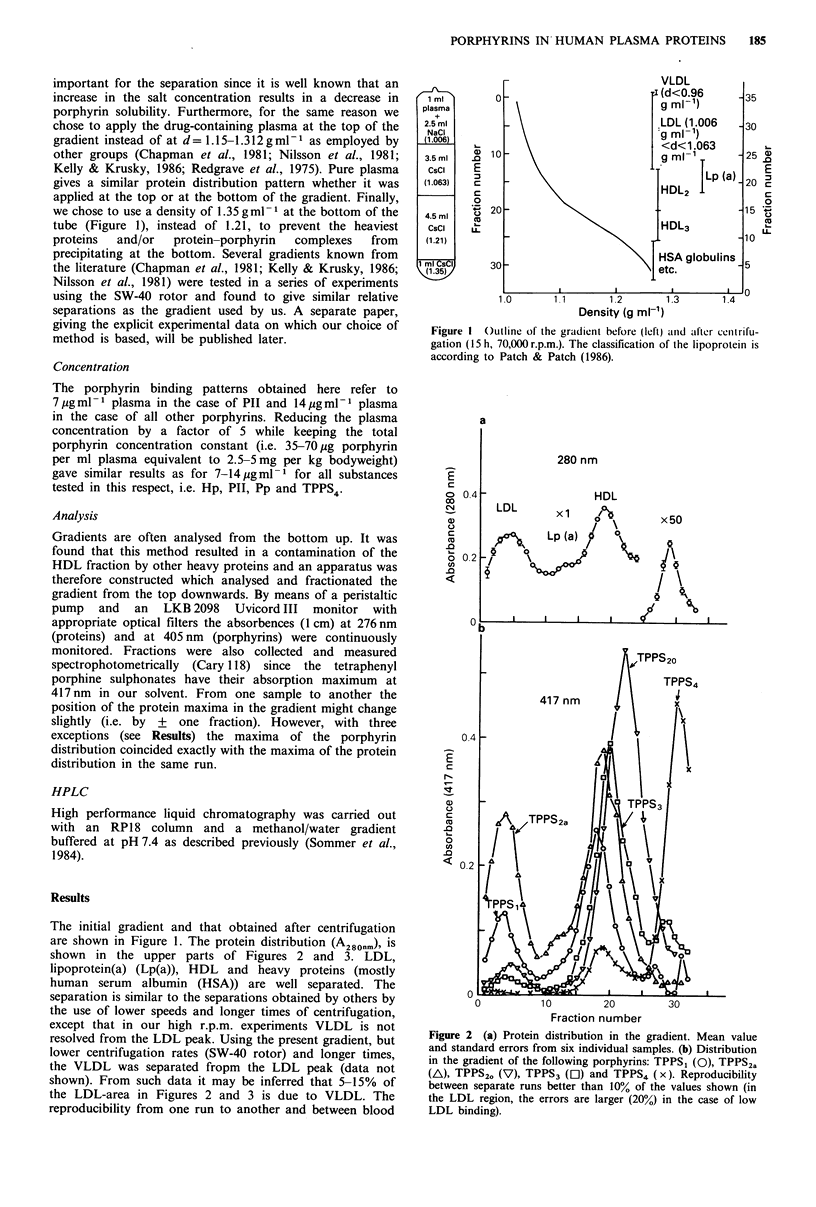

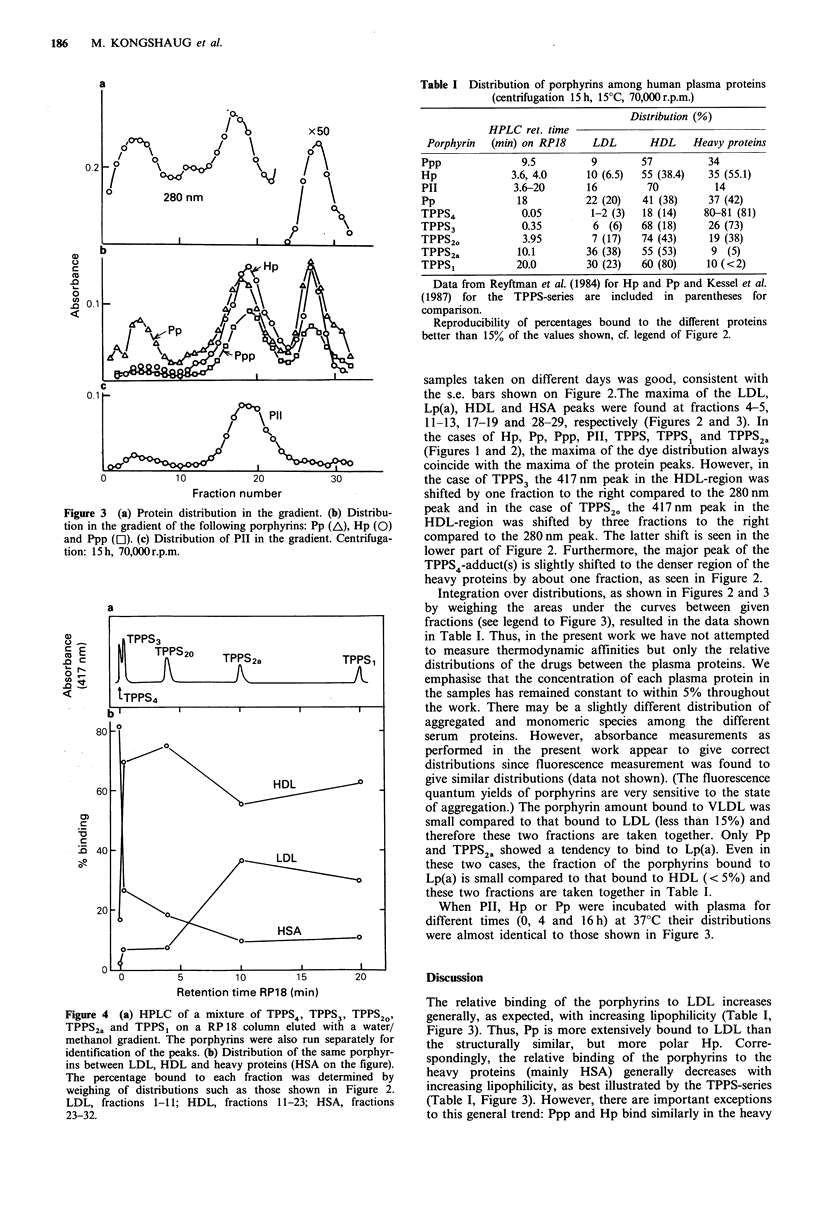

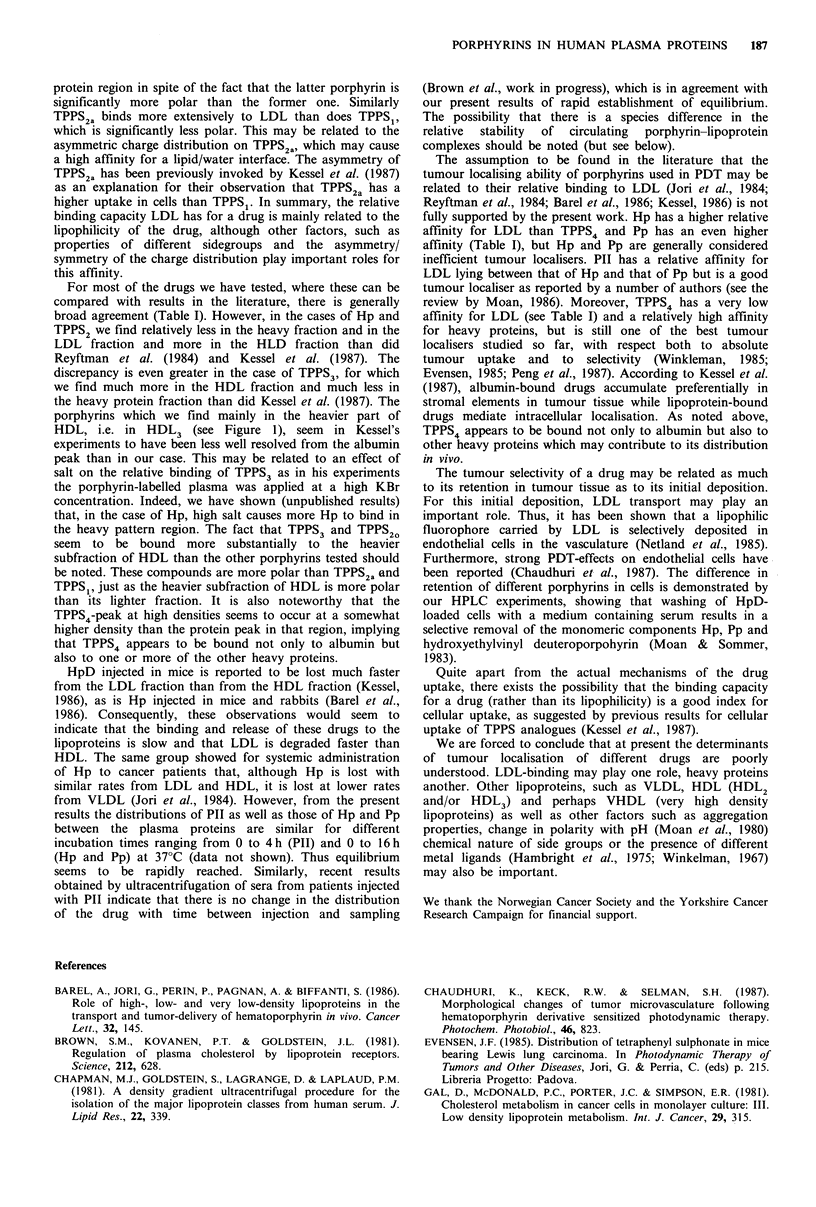

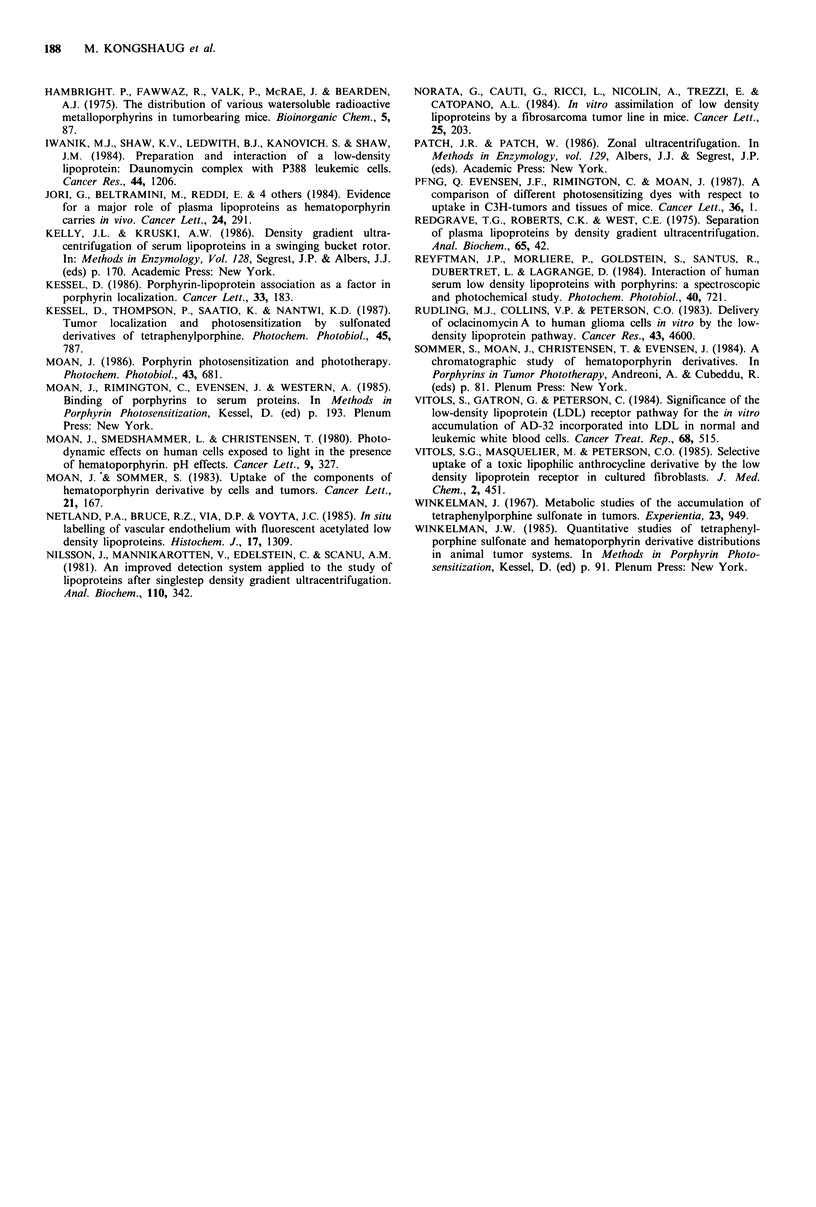


## References

[OCR_00586] Barel A., Jori G., Perin A., Romandini P., Pagnan A., Biffanti S. (1986). Role of high-, low- and very low-density lipoproteins in the transport and tumor-delivery of hematoporphyrin in vivo.. Cancer Lett.

[OCR_00592] Brown M. S., Kovanen P. T., Goldstein J. L. (1981). Regulation of plasma cholesterol by lipoprotein receptors.. Science.

[OCR_00597] Chapman M. J., Goldstein S., Lagrange D., Laplaud P. M. (1981). A density gradient ultracentrifugal procedure for the isolation of the major lipoprotein classes from human serum.. J Lipid Res.

[OCR_00603] Chaudhuri K., Keck R. W., Selman S. H. (1987). Morphological changes of tumor microvasculature following hematoporphyrin derivative sensitized photodynamic therapy.. Photochem Photobiol.

[OCR_00615] Gal D., MacDonald P. C., Porter J. C., Simpson E. R. (1981). Cholesterol metabolism in cancer cells in monolayer culture. III. Low-density lipoprotein metabolism.. Int J Cancer.

[OCR_00622] Hambright P., Fawwaz R., Valk P., McRae J., Bearden A. J. (1975). The distribution of various water soluble radioactive metalloporphyrins in tumor bearing mice.. Bioinorg Chem.

[OCR_00628] Iwanik M. J., Shaw K. V., Ledwith B. J., Yanovich S., Shaw J. M. (1984). Preparation and interaction of a low-density lipoprotein:daunomycin complex with P388 leukemic cells.. Cancer Res.

[OCR_00634] Jori G., Beltramini M., Reddi E., Salvato B., Pagnan A., Ziron L., Tomio L., Tsanov T. (1984). Evidence for a major role of plasma lipoproteins as hematoporphyrin carriers in vivo.. Cancer Lett.

[OCR_00639] Kelley J. L., Kruski A. W. (1986). Density gradient ultracentrifugation of serum lipoproteins in a swinging bucket rotor.. Methods Enzymol.

[OCR_00645] Kessel D. (1986). Porphyrin-lipoprotein association as a factor in porphyrin localization.. Cancer Lett.

[OCR_00649] Kessel D., Thompson P., Saatio K., Nantwi K. D. (1987). Tumor localization and photosensitization by sulfonated derivatives of tetraphenylporphine.. Photochem Photobiol.

[OCR_00655] Moan J. (1986). Porphyrin photosensitization and phototherapy.. Photochem Photobiol.

[OCR_00659] Moan J., Rimington C., Evensen J. F., Western A. (1985). Binding of porphyrins to serum proteins.. Adv Exp Med Biol.

[OCR_00665] Moan J., Smedshammer L., Christensen T. (1980). Photodynamic effects on human cells exposed to light in the presence of hematoporphyrin. pH effects.. Cancer Lett.

[OCR_00670] Moan J., Sommer S. (1983). Uptake of the components of hematoporphyrin derivative by cells and tumours.. Cancer Lett.

[OCR_00675] Netland P. A., Zetter B. R., Via D. P., Voyta J. C. (1985). In situ labelling of vascular endothelium with fluorescent acetylated low density lipoprotein.. Histochem J.

[OCR_00680] Nilsson J., Mannickarottu V., Edelstein C., Scanu A. M. (1981). An improved detection system applied to the study of serum lipoproteins after single-step density gradient ultracentrifugation.. Anal Biochem.

[OCR_00686] Norata G., Canti G., Ricci L., Nicolin A., Trezzi E., Catapano A. L. (1984). In vivo assimilation of low density lipoproteins by a fibrosarcoma tumour line in mice.. Cancer Lett.

[OCR_00701] Redgrave T. G., Roberts D. C., West C. E. (1975). Separation of plasma lipoproteins by density-gradient ultracentrifugation.. Anal Biochem.

[OCR_00706] Reyftmann J. P., Morliere P., Goldstein S., Satus R., Dubertret L., Lagrange D. (1984). Interaction of human serum low density lipoproteins with porphyrins: a spectroscopic and photochemical study.. Photochem Photobiol.

[OCR_00712] Rudling M. J., Collins V. P., Peterson C. O. (1983). Delivery of aclacinomycin A to human glioma cells in vitro by the low-density lipoprotein pathway.. Cancer Res.

[OCR_00729] Vitols S. G., Masquelier M., Peterson C. O. (1985). Selective uptake of a toxic lipophilic anthracycline derivative by the low-density lipoprotein receptor pathway in cultured fibroblasts.. J Med Chem.

[OCR_00723] Vitols S., Gahrton G., Peterson C. (1984). Significance of the low-density lipoprotein (LDL) receptor pathway for the in vitro accumulation of AD-32 incorporated into LDL in normal and leukemic white blood cells.. Cancer Treat Rep.

[OCR_00735] Winkelman J. (1967). Metabolic studies on the accumulation of tetraphenylporphinesulfonate in tumours.. Experientia.

